# Validation of diagnostic utility of fasting plasma glucose and HbA1c in stable renal transplant recipients one year after transplantation

**DOI:** 10.1186/s12882-018-1171-3

**Published:** 2019-01-10

**Authors:** Amin M. Ussif, Anders Åsberg, Thea Anine Strøm Halden, Espen Nordheim, Anders Hartmann, Trond Jenssen

**Affiliations:** 1 Department of Transplantation Medicine, Oslo University Hospital, Rikshospitalet, P.O.Box 4950, 0424 Oslo, Nydalen Norway; 2 Department of Pharmaceutical Biosciences, School of Pharmacy, University of Oslo, Oslo, Norway; 3 The Norwegian Renal Registry, Oslo University Hospital, Rikshospitalet, Oslo, Norway; 4 Institute of Clinical Medicine, Faculty of Medicine, University of Oslo, Oslo, Norway; 5 Metabolic and Renal Research Group, Faculty of Health Sciences UiT, The Arctic University of Norway, Tromsø, Norway

**Keywords:** Renal transplantation, Post-transplantation diabetes mellitus, Diagnosis, Oral glucose tolerance test, HbA1c

## Abstract

**Background:**

The use of HbA1c ≥6.5% for diagnosis of diabetes has been challenged for post-transplantation diabetes mellitus (PTDM) also known as new onset diabetes after transplantation (NODAT) due to a low sensitivity early after renal transplantation. PTDM diagnosed with an oral glucose tolerance test (OGTT) is highly predictable for long-term patient mortality. HbA1c was introduced for diagnosis based on the risk of developing diabetic retinopathy. The utility of HbA1c measures versus glucose criteria has not been widely assessed in stable transplant patients but still HbA1c is widely used in this population. The aim of the present analyses was to validate the utility of fasting plasma glucose (FPG) together with HbA1c in diagnosing PTDM in stable renal transplant recipients (RTRs).

**Methods:**

OGTT’s were performed one year after transplantation in 494 consecutive RTRs without diabetes. FPG and HbA1c were obtained the same day, before starting the OGTT. Validation was performed using C-statistics and logistic regression analyses.

**Results:**

PTDM was diagnosed in 51 patients (10.3%) by glucose criteria, 38 (74%) patients were diagnosed by FPG ≥7.0 mmol/L [126.1 mg/dl], and 13 (26%) only by 2-h plasma glucose. Six of the latter had HbA1c ≥6.5%. Only seven patients out of the 51 (13.7%) PTDM patients remained undiagnosed when HbA1c ≥6.5% was used together with FPG, and five of these regressed to normal after a median follow-up of 14 months. ROC curves including FPG and HbA1c versus OGTT derived criteria revealed an AUC of 0.858.

**Conclusions:**

Combining standard diagnostic FPG and HbA1c criteria captured almost all patients with persistent PTDM in stable RTRs. The combined use of the criteria appears to be an applicable diagnostic strategy for PTDM without the need of an OGTT one year post-transplant.

**Trial registration:**

Retrospectively registered.

## Background

Post-transplantation diabetes mellitus (PTDM) is a term for diabetes that is diagnosed after solid organ transplantation. The diagnosis has traditionally been based on glucose criteria according to an oral glucose tolerance test (OGTT) [1]. However, with the introduction of HbA1c ≥6.5% as a diagnostic measure for type 2 diabetes [2, 3] questions have been raised regarding the use of this criterion also for PTDM, at least after renal transplantation [4–6]. While the PTDM diagnosis made by the glucose criterion primarily defines increased mortality risk for the patient [7], the HbA1c criterion in type 2 diabetes is chosen merely according to the risk of developing diabetic retinopathy [8]. Other arguments against the use of HbA1c are particularly relevant to the early phase following renal transplantation with changes in erythropoiesis and introduction of anti-proliferative immunosuppressive drugs amongst other interacting factors on HbA1c [4, 6]. In agreement with these notions a previous study of early PTDM after renal transplantation revealed that the sensitivity of HbA1c ≥6.5% was as low as 50% for the diagnosis of PTDM [9]. When the glucose criteria during OGTT for the diagnosis of PTDM were used, we confirmed previous findings that PTDM had a detrimental effect on long-term cardiovascular outcomes, but HbA1c ≥6.5% per se did not significantly associate with adverse outcomes [7]. Other investigators have argued that a cut-off value for HbA1c ≥6.2% may be reasonable for diagnosis of PTDM in the early phase after transplantation [10]. A combination of the HbA1c criteria and OGTT in high risk patients may be another approach as advocated by an international consensus meeting on PTDM [11]. However, there is probably need for a simplified strategy in daily clinical routine.

It is conceivable that HbA1c associates better with glucose long-term than the first months after transplantation. One year after transplantation hemoglobin values are usually normalized and stable in successfully RTRs. During the last few years we have examined almost RTRs at 1 year after transplantation, and included OGTTs for PTDM in patients who did not have a diagnosis of PTDM at this time point. The aims of this study were to examine whether the HbA1c criteria were useful for the diagnosis of PTDM and whether a combination of FPG and HbA1c drawn in a single fasting state could be used for diagnosis without need for an OGTT in a stable phase after renal transplantation.

## Methods

**Fig. 1:**
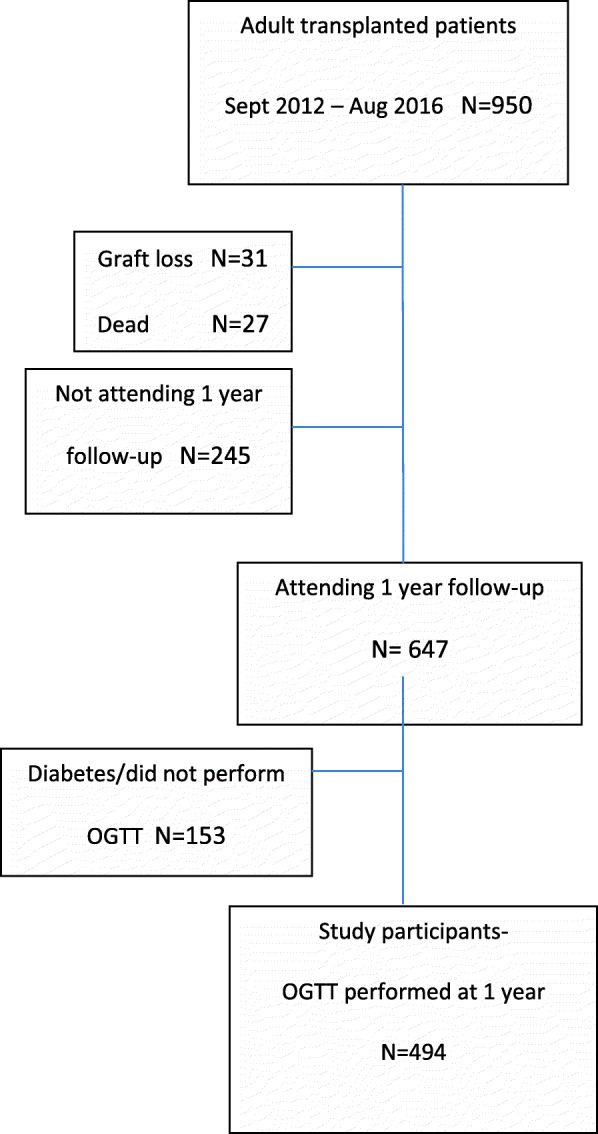
Patient disposition chart

All renal transplantations in Norway are performed at the National Transplant center in Oslo. As part of the routine follow-up most patients return to the transplant center after 1 year for thorough investigations including an OGTT. Only patients without prior diagnosis of diabetes or PTDM undergo the glucose challenge test at that time. In the time period between September 2012 and August 2016 a total of 950 patients over 18 years of age were transplanted and 647 patients were attending 1 year follow-up. Altogether 494 patients without diabetes underwent testing with valid results from the OGTT 1 year after transplantation. The disposition of the patients is shown in Fig. 1.

The immunosuppressive protocol consisted of basiliximab (20 mg iv on day 0 and 4) and methylprednisolone (250/500 mg iv on day 0 in standard/high risk patients) induction, followed by tacrolimus, mycophenolate mofetil and prednisolone maintenance. Oral tacrolimus was initiated at the day of transplantation, starting with 0.04 mg/kg twice daily in standard risk patients and 0.05 mg/kg twice daily in high risk patients. TDM was applied and doses were adjusted to reach target whole blood trough concentrations of 3 to 7 μg/L in standard immunological risk patients, and 8 to 12 μg/L (days 0–30) followed by 6 to 10 μg/L (after day 30) in high immunological risk patients. The mycophenolate mofetil dose was 750 mg twice daily and prednisolone was given once daily, 20 mg on day 1 (80 mg in high risk patients), and tapered to 10 mg at 4 weeks in standard risk and 8 weeks in high risk patients.

Clinical and laboratory data were retrospectively retrieved from the clinical biobank and the Norwegian Renal Registry. All patients had signed an informed consent for use of the data for research purposes concerning outcomes after renal transplantation and the Regional Ethics Committee for South-East Norway had approved studies on outcomes in this population.

### Statistical methods

Characteristics of the sample population were presented and subgroups were compared based on glucose criteria. Differences within the three subgroups were tested using the ANOVA test for continuous variables and the Chi-square test for categorical variables. Logistic regression was conducted using HbA1c and FPG as the covariates vs 2hPG (OGTT) as the dependent variable.

Furthermore, ROC curve analysis was conducted and sensitivity, specificity, positive predictive value (PPV) and negative predictive value (NPV) calculated. All analyses were conducted using IBM SPSS release 24.0.0.1 and R version 3.4.3 [12].

## Results

Demographic data of the study population and subgroups according to glucose tolerance; normal glucose tolerance (NGT), impaired glucose tolerance (IGT) and diabetes (PTDM) are shown in Table 1. Two thirds of the recipients were males with ages ranging from 18 to 82 years. Almost a third of the patients had living donors.

**Table 1:** Demographic and clinical characteristics of 494 patients by glucose tolerance subgroups

	All patients	NGT	IGT	PTDM	*P*-value
Number of patients	494	377	66	51	
Age (years)	52.5 ± 14.0	50.5 ± 14.2	58.1 ± 10.8	60.6 ± 11.4	< 0.001
Donor (% living)	152 (30.8)	121 (32.1)	22 (33.3)	9 (17.6)	0.10
Gender (% males)	343 (69.2)	251 (66.6)	51 (77.3)	39 (76.5)	0.11
BMI (kg/m^2^)	25.9 ± 4.5	25.5 ± 4.4	26.4 ± 4.5	27.4 ± 5.1	0.01
Hemoglobin (g/dL)	13.7 ± 1.7	13.7 ± 1.7	13.7 ± 1.7	13.8 ± 1.6	0.83
HbA1c (%)	5.6 ± 0.5	5.5 ± 0.4	5.9 ± 0.4	6.4 ± 0.7	< 0.001
Fasting plasma glucose					
(mmol/L)	5.6 ± 0.9	5.4 ± 0.5	5.8 ± 0.5	7.4 ± 1.0	< 0.001
[mg/dL]	[100.9 ± 16.2]	[97.3 ± 9.0]	[104.5 ± 9.0]	[133.3 ± 18.0]	
2 h plasma glucose after OGTT					
(mmol/L)	6.7 ± 2.5	5.6 ± 1.1	8.9 ± 0.9	11.3 ± 3.6	< 0.001
[mg/dL]	[117.1 ± 45.0]	[100.9 ± 19.8]	[160.4 ± 16.2]	[203.6 ± 64.9]	
Creatinine (μmol/L)	117 ± 40	115 ± 38	125 ± 46	118 ± 45	0.20
Total cholesterol					
(mmol/L)	4.9 ± 1.0	4.8 ± 1.0	5.1 ± 1.1	5.0 ± 1.0	0.10
[mg/dL]	[88.3 ± 18.0]	[86.5 ± 18.0]	[91.9 ± 19.8]	[90.1 ± 18.0]	
LDL-cholesterol					
(mmol/L)	2.9 ± 0.9	2.8 ± 0.8	3.0 ± 0.9	2.9 ± 0.9	0.17
[mg/dL]	[52.3 ± 16.2]	[50.5 ± 14.4]	[54.1 ± 16.2]	[52.3 ± 16.2]	
Triglycerides					
(mmol/L)	1.6 ± 0.8	1.5 ± 0.8	1.8 ± 1.0	2.0 ± 1.0	< 0.001
[mg/dL]	[28.8 ± 14.4]	[27.0 ± 14.4]	[32.4 ± 18.0]	[36.0 ± 18.0]	

Difference between subgroups given in mean ± standard deviation or number (%), evaluated by the ANOVA test for continuous variables and Pearson chi-square test for categorical variables. *P*-values are reported to 2 decimals. Abbreviations: BMI, body mass index in kg/m2; *IGT* impaired glucose tolerance, *LDL* low density lipoprotein, *NGT* normal glucose tolerance, *OGTT* oral glucose tolerance test, *PTDM* post-transplantation diabetes mellitus

### Diagnosis of PTDM by glucose criteria

Table 1 reveals the differences between the glucose tolerance groups based on the OGTT. There was a significant difference in age between the groups, the PTDM patients were 10 years older than NGT patients and had slightly higher BMI. Beyond the difference in glucose measures also HbA1c and lipid profiles were different between the groups. A total of 51 patients (10.3%) were diagnosed with PTDM by the glucose criteria.

**Fig. 2:**
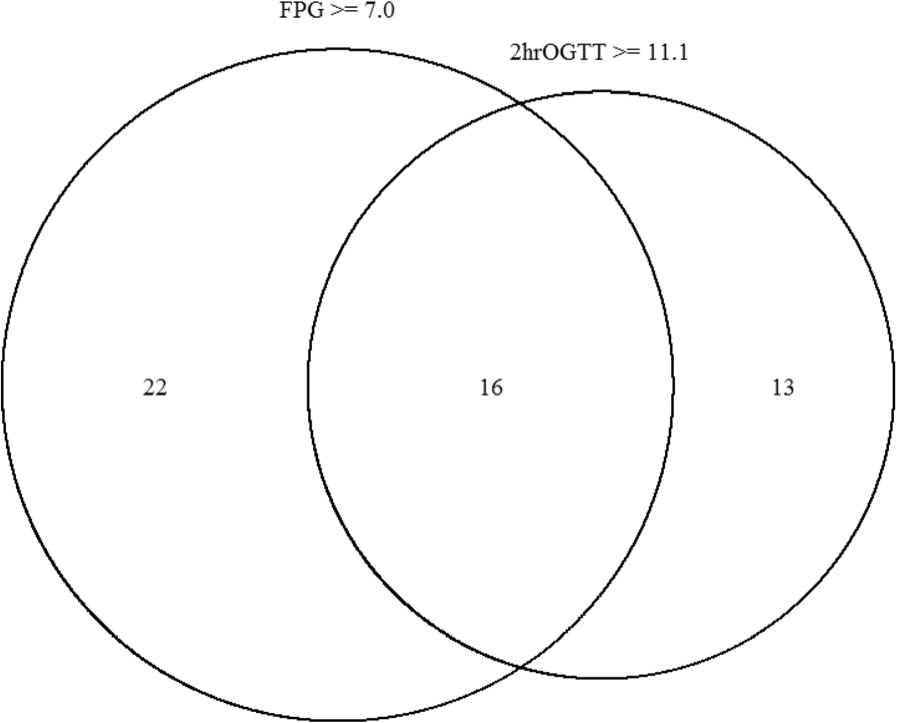
Venn diagram illustrating the overlap between FPG and 2hPG as diagnostic findings in 51 PTDM patients

The diagnosis of PTDM was obtained by FPG ≥7.0 mmol/L [126.1 mg/dL] in 38 out of 51 patients (74%). The remaining 13 patients that had FPG < 7.0 mmol/L [126.1 mg/dL] were diagnosed by isolated 2-h plasma glucose (2hPG) ≥11.1 mmol/L [200.0 mg/dL] following OGTT. The overlap of glucose criteria in the PTDM patients is illustrated in Fig. 2. Among those with PTDM 21 (41%) had HbA1c ≥6.5% and 30 (59.0%) had HbA1c ≥6.2%.

Sixty-six (13.4%) patients were diagnosed with IGT and among those 443 without diabetes 5 (1.1%) had HbA1c ≥6.5% and 18 (4.0%) had HbA1c ≥6.2%).

### Diagnosis of PTDM by HbA1c criteria

Table 2 shows the sensitivity, specificity, positive- and negative predictive values for different cut-off values of HbA1c for the diagnosis of PTDM. The sensitivity for the HbA1c criteria for a diagnosis of PTDM was 43% for HbA1c ≥6.5 and 69% for HbA1c ≥6.2%. However, the specificity of the diagnosis was 97% with HbA1c ≥ 6.5% versus 88% for HbA1c ≥6.2%. When patients with a diagnosis of PTDM by FPG (*n* = 38) were taken out of the analysis the data remained similar (sensitivity was 46% for HbA1c ≥6.5 and 69% for HbA1c ≥6.2%., specificity of the diagnosis was 98% with HbA1c ≥ 6.5% versus 90% for HbA1c ≥6.2%).

**Table 2:** Diagnostic performance of HbA1c at different cut-off values in all 494 patients

	Sensitivity (%)	Specificity (%)	PPV (%)	NPV (%)
HbA1c ≥5.5%	88.0	44.5	15.4	97.0
HbA1c ≥6.0%	79.4	81.8	33.4	97.2
HbA1c ≥6.2%	69.0	88.1	40.0	96.1
HbA1c ≥6.5%	43.1	97.2	63.9	93.7

Abbreviations: *NPV* negative predictive value, *PPV* positive predictive value

### Combination of fasting plasma glucose and HbA1c criteria

**Fig. 3:**
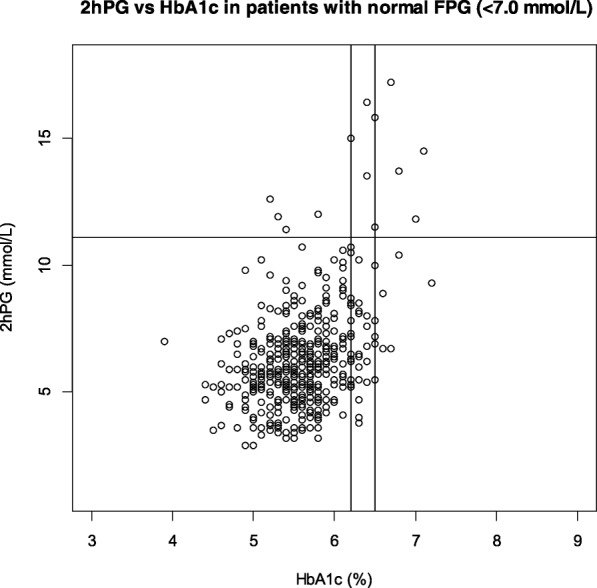
Diagram illustrating the 2-h plasma glucose versus HbA1c values in all 456 patients after removal of 38 patients diagnosed with PTDM using FPG criteria

The relationship between HbA1c and 2hPG after OGTT in patients with normal FPG (< 7.0 mmol/L) [126.1 mg/dL] is shown in Fig. 3. This figure shows the additional value of HbA1c for the diagnosis of PTDM, beyond what FPG provide by itself. As seen in the upper left quadrant, seven patients remained undiagnosed when HbA1c ≥6.5% was used as cut-off. When FPG ≥7.0 mmol/L [126.1 mg/dL] and HbA1c ≥6.5% were combined, 44 out of 51 (86%) PTDM patients were diagnosed. We examined the trajectories of glucose values for these seven “false negative” patients that showed an isolated 2hPG ≥11.1 mmol/L [200.0 mg/dL]; five had a median FPG of 5.8 (range 5.7–6.1) mmol/L [102.7–109.9 mg/dL] and median HbA1c of 5.6 (range 5.2–6.0) % after a median observation time of 14 months (range 3–40). The two others received oral antidiabetic medication after a mean observation time of 18 months. Eleven patients had HbA1c ≥ 6.5% without fulfilling the OGTT criteria. Five of these had IGT and only one had a completely normal FPG.

The sensitivity was improved when the cut-off value was lowered to HbA1c ≥6.2%. However, the number of false positive diagnosis of PTDM increased manifold as apparent from the lower right quadrant in Fig. 3.

**Fig. 4:**
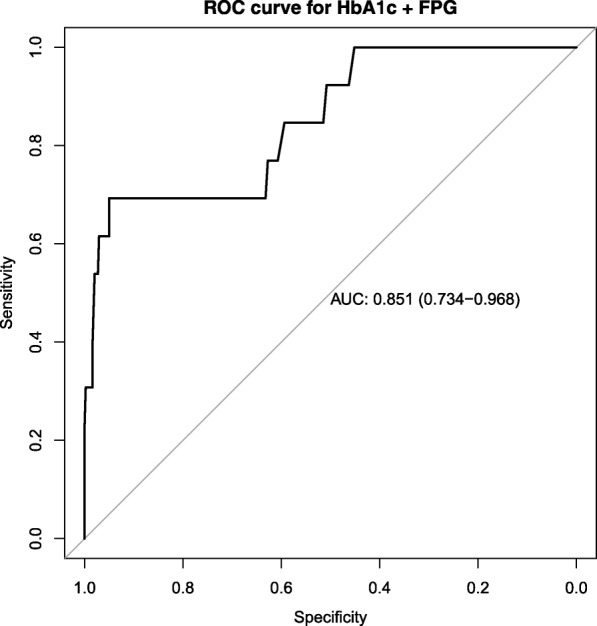
Combined ROC curve fasting plasma glucose and HbA1c as predictors. Logistic regression coefficients for HbA1c and FPG with *p*-values are β = 2.24(0.001) and 1.82(0.007) respectively

**Fig. 5:**
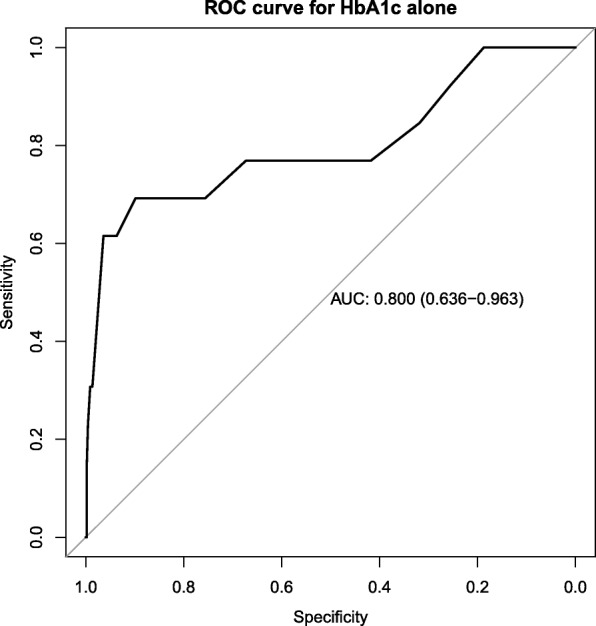
ROC curve for HbA1c alone as predictor

To further demonstrate the utility of our results we conducted a multivariate ROC analysis as shown in Fig. 4. Combining information from both FPG and HbA1c variables in the logistic regression on a patient improved the AUC from 0.800 (see Fig. 5) to 0.856. That is the discriminatory power has improved by 5.6%.

## Discussion

The novel aspect of the present study is that the standard diagnostic criteria with FPG ≥7.0 mmol/L [126.1 mg/dL] used in combination with HbA1c ≥6.5% allows for a simplified diagnostic strategy that captures almost all patients with persisting PTDM in a stable phase 1 year after renal transplantation. Comprising almost 500 patients, this study is to our knowledge the biggest addressing the use of HbA1c as a supplementary diagnostic tool together with FPG. When this approach was used, seven out of 51 patients were missed, but upon further follow-up of these patients five regressed to normal glucose tolerance after 14 months. Only two patients had persisting PTDM and later received oral antidiabetic medication. On the other hand; eleven false positive patients were diagnosed with PTDM when the HbA1c criterion was used, as they showed 2hPG < 11.1 mmol/L [200.0 mg/dL] following OGTT. This may not be a challenge since they initially would not need drugs or any particular counseling beyond normal practice. When the cut-off was lowered to HbA1c ≥6.2%, in accordance to what has also been advocated for PTDM in an early phase after transplantation [10], the sensitivity was slightly higher but the number of false negatives increased manifold. Again we have demonstrated the application of ROC analysis to two quantitative tests that is HbA1c and FPG in this paper. The model can further be extended to include additional variables such as age and other demographic factors.

The limitations for use of HbA1c for the diagnosis of PTDM have been discussed extensively in the literature [4, 5, 9, 10]. Different recommendations have been put forward for PTDM, such as a combination of HbA1c criteria and OGTT in high risk patients [11], random plasma glucose measurements [13] or OGTT with slightly elevated FPGs > 5.1 mmol/L [91.9 mg/dL] [14, 15]. The idea with the present study was to evaluate an even simpler strategy that did not include new tests, new algorithms or altered practice but simply evaluate the utility of the combined diagnostic criteria in a single fasting blood sample. There is probably no single strategy without using an OGTT that can allow for a correct diagnosis of all patients with PTDM even in a stable phase after renal transplantation. The most important issue is of course the trajectory of the disease and finally the outcomes for the patients. With the present strategy we found that five of the patients who missed a diagnosis had regressed to normal glucose tolerance after more than 1 year of observation. Using a lower HbA1c cut-off would retrieve more PTDM patients but a substantial number of patients would then have a false diagnosis that may not feel appropriate. With the generally recommended HbA1c limit of 6.5% a persistent PTDM diagnosis was only missed in two out of 494 patients that underwent OGTT. However, we could not evaluate the trajectory for patients with IGT that are potentially at risk for future PTDM.

### Strengths and weaknesses

Our study validated a simple strategy for the diagnosis of PTDM in RTRs in a stable phase 1 year after transplantation using a single fasting blood sample without routine use of more cumbersome OGTT which has been the gold standard. The group of patients examined is large compared to previous studies from other groups and representative as for a nationwide cohort of patients. Data have also been sampled consecutively in all patients using a similar protocol.

The number of PTDM patients is relatively low for firm conclusions. More than 90% were Caucasians and therefore external validation of the data is warranted.

## Conclusions

Combining standard diagnostic FPG and HbA1c criteria captured almost all patients with persistent PTDM in stable RTRs. The combined use of the criteria appears to be an applicable diagnostic strategy for PTDM without the need of an OGTT 1 year posttransplant.

## Abbreviations

2hPG: 2 h plasma glucose
ADA: American Diabetes Association
FPG: Fasting plasma glucose
HbA1c: Glycated hemoglobin
IGT: Impaired glucose tolerance
NGT: Normal glucose tolerance
NPV: Negative predictive value
OGTT: Oral glucose tolerance test
PPV: Positive predictive value
PTDM: Post-transplantation diabetes mellitus
RTRs: Renal transplant recipients
WHO: World Health Organization
